# Divergence in virus-host protein interactomes across species explains disparities in translational therapeutic success

**DOI:** 10.1128/mbio.00195-26

**Published:** 2026-06-22

**Authors:** Kang Tang, Kai Zhuang, Zuyi Zhao, Bingsong Zhang, Xin Liu, Shisi Li, Jing Tang, Yilin Chen, Xiangjun Du

**Affiliations:** 1 School of Public Health, Guangdong Medical University, Dongguan, People's Republic of China; 2 School of Public Health (Shenzhen), Sun Yat-Sen University, Guangzhou, People's Republic of China; 3 School of Public Health (Shenzhen), Shenzhen Campus of Sun Yat-sen University, Shenzhen, People's Republic of China; 4 Laboratory of Mathematics and Complex Systems, School of Mathematical Sciences, Beijing Normal University, Beijing, People's Republic of China; 5 Bao'an District Hospital for Chronic Diseases Prevention and Cure 117846, Shenzhen, People's Republic of China; 6 School of Biomedical Engineering, Guangdong Medical University, Dongguan, People's Republic of China; 7 Shenzhen Key Laboratory of Pathogenic Microbes & Biosafety, Shenzhen Campus of Sun Yat-sen University, Shenzhen, People's Republic of China; 8 Key Laboratory of Tropical Disease Control, Ministry of Education, Sun Yat-sen University, Guangzhou, People's Republic of China; Tsinghua University, Beijing, China

**Keywords:** interactome, virus-host interaction, network resilience, therapeutic approval

## Abstract

Infectious viral diseases remain a major and persistent threat to human health, and findings from animal models often translate poorly to clinical applications. Here, we constructed protein interactomes for seven species, including humans, revealing substantial differences in research biases. A random sampling strategy was employed to generate human interactomes, with sizes matched to those of the other six species to ensure cross-species comparability. We found that the clustering of virally targeted proteins within the interactomes (measured as largest connected component [LCC] proportion) and network fragmentation after their removal correlated with network density. Viral perturbations in species other than humans were heterogeneous, but generally showed reduced clustering and increased network fragments. The relative degree of network fragmentation (quantified by relative IC values) was correlated with local network conservation rather than sequence similarity, indicating that local network structure is selected, maintaining resilience during virus–host interactions. Furthermore, relative IC values and LCC proportion differences were positively associated with non-vaccine therapeutic success rates from phase I trials to approval. Our findings present a novel paradigm for the comparative analysis of disease phenotypes across species, thereby providing a new evaluative metric for selecting animal models in translational biomedical research.

**IMPORTANCE:**

Cross-species network comparisons help uncover the molecular mechanisms of complex phenotypes. The viral module formation within interactomes and the network resilience during viral infection are of key impacts on hosts. The analysis of the interactomes of seven species shows that network density critically influences the network metrics performance. Under size-matched conditions, non-human species exhibited more complex viral perturbations than humans, but with smaller viral module size and more network fragmentation. The difference in fragmentation had a positive correlation with the conservation of the local network structure of virally targeted proteins, implying that viral perturbations act as evolutionary signals at the network level. Additionally, differences in fragmentation and viral module size were positively associated with the rates of successful progression from phase I trials to approval. This work offers a new framework for cross-species disease analysis and guides model organism selection for viral infection research.

## INTRODUCTION

Despite advances in medical technology and sanitation have led to a reduction in the infectious diseases burden, climate change, demographic shifts, and increasing global travel continue to engender recurrent endemic and emerging diseases, posing serious health and socioeconomic threats (1). Owing to their rapid mutation rates, viral pathogens can evade immunity and trigger epidemics. Recent examples include the SARS-CoV-2-driven COVID-19 pandemic (2), the global mpox outbreak in 2022 (3), and the persistence of chikungunya in tropical and subtropical regions (4). These challenges underscore the urgent need for effective therapeutic strategies against viral infections.

A survey of infectious disease clinical trials over the past decade showed that only 13.2% of therapeutic candidates progressed from phase I to approval (5). Laboratory mice (*Mus musculus*), the most widely used animal model, share a common mammalian ancestor with humans, with over 14,000 1:1 orthologous protein-coding genes conserved between the two species (6). This genetic similarity supports the feasibility of using mouse models in biomedical research but also masks translational challenges, often leading to clinical trial failures. For example, fibrotic complications, which are observed in approximately one-third of patients with Crohn’s disease, are particularly challenging to faithfully recapitulate in mouse models (7). These discrepancies stem from ~90 million years of independent evolution in distinct ecological niches, leading to substantial interspecies differences. Evolutionary approaches can help elucidate these divergence patterns and pave the way for the development of more translatable biomedical research frameworks.

Viral perturbations and infection establishment constitute complex biological phenotypes. Conventional reductionist approaches, which focus on a limited number of molecules in specific pathways, often fail to fully explain these mechanisms, restricting clinical translation. Conversely, systems biology focuses on genotype-phenotype relationships through dynamic interactions among genes and their products (8). These interactions can be modeled as networks, wherein biological entities such as proteins, DNA sequences, and metabolites are represented as nodes, and their physical or functional relationships are represented as edges (9).

The aim of evolutionary systems biology is to clarify how evolutionary processes shape genomes, molecular networks, and phenotypes (7). Comparative analyses between animal models and humans can be used to study evolutionary conservation or divergence in underlying molecular networks. Using a network-based metric, Zitnik et al. showed that the enhanced resilience of protein-protein interaction (PPI) networks correlated with an organism’s ability to thrive in complex and dynamic environments (10). The rapid accumulation of high-throughput interactome data, together with advances in systems biology, has driven increasing interest in network-based approaches to studying viral infectious diseases (11–13). This perspective offers a holistic framework for understanding disease mechanisms. However, few studies have examined molecular network stability under viral perturbation from an evolutionary systems perspective, which could yield critical insight into phenotypic differences in viral infection between traditional animal models and humans.

**Fig 1:**
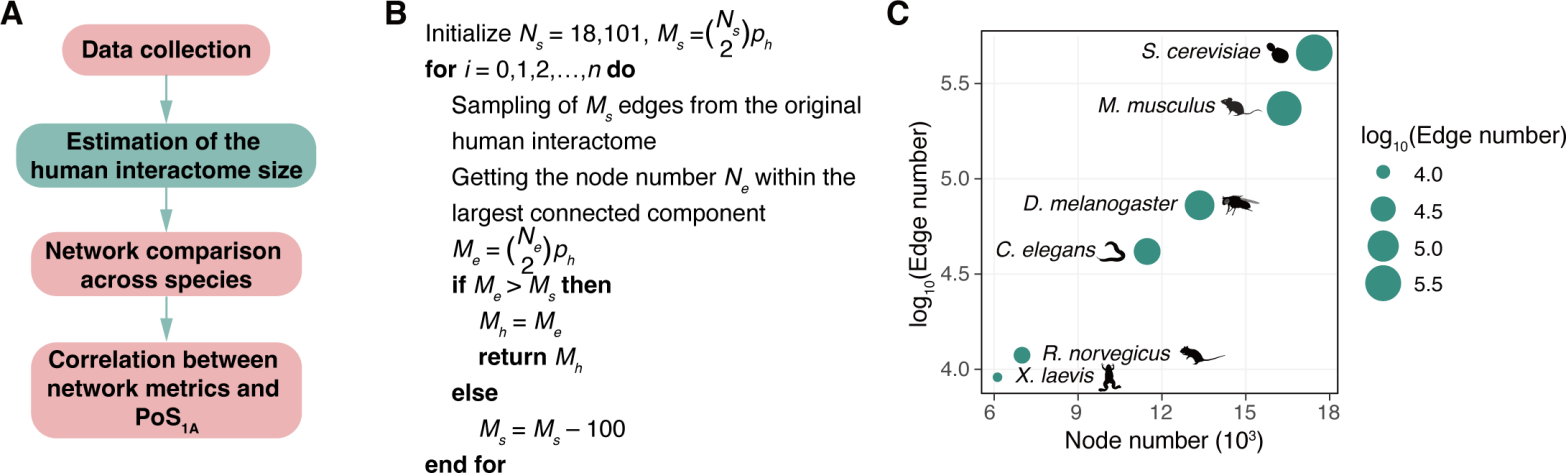
Estimation of the human interactome size. (**A**) Overview of network-based framework for analysis of evolutionary divergence in virus-host interactions. (**B**) Describing the process of human interactome size estimation. (**C**) Human interactome size estimated by comparison to different species with respect to the number of protein-coding genes and PPIs. The circle area is proportional to the PPI number. *N_s_*, the number of starting nodes in the human interactome; *M_s_*, the edge number in the human interactome corresponding to *N_s_* = 18,101 nodes; *N_e_*, the node number within the largest connected component; *M_e_*, the edge number in the human interactome corresponding to *N_e_* nodes; *p_h_*, the probability of each possible PPI in humans; PPI, protein-protein interaction.

In this study, we integrated PPIs from multiple databases and used a random sampling strategy to generate human interactomes that matched in network size to those of six comparator species (Fig. 1). We then compared the aggregation levels of virally targeted proteins within the human interactome and their orthologs in the corresponding species-specific interactomes, as well as the relative extent of network fragmentation following the removal of these targets from each network. To explore the stability of network architectures, we defined a local network structural conservation metric. Finally, we assessed how sequence divergence and network structural differences relate to the translational utility of mouse models and the success of antiviral therapeutics. These findings illuminate species-specific viral perturbations and inform rational animal model selection.

## RESULTS

### The protein interactome landscape of the seven species

We integrated data from five major PPI databases and one recent study to explore the evolutionary patterns from a network biology view, ultimately selecting seven well-studied model organisms. The largest connected component of human (*Homo sapiens*), mouse (*Mus musculus*), rat (*Rattus norvegicus*), African clawed frog (*Xenopus laevis*), fruit fly (*Drosophila melanogaster*), *Caenorhabditis elegans*, and yeast (*Saccharomyces cerevisiae*) protein interactomes contained 18,101, 11,297, 2,837, 1,131, 8,859, 7,502, and 4,982 proteins, along with 698,482, 187,081, 5,766, 1,528, 56,260, 32,411, and 151,766 PPIs, respectively (Table 1).

**TABLE 1:** Network properties of protein interactomes in seven species

Species	Number of nodes *N*	Number of edges *M*	Mean degree <*k*>	Mean clustering coefficient <*c*>	Mean distance <*d*>	Diameter *d*_max_	Density
*H. sapiens*	18,101	698,482	77.18	0.12	2.73	6	0.00,426
*M. musculus*	11,297	187,081	33.12	0.22	3.43	12	0.00,293
*R. norvegicus*	2,837	5,766	4.06	0.12	3.54	12	0.00,143
*X. laevis*	1,131	1,528	2.70	0.07	3.25	10	0.00,239
*D. melanogaster*	8,859	56,260	12.70	0.10	3.80	10	0.00,143
*C. elegans*	7,502	32,411	8.64	0.12	4.28	13	0.00,115
*S. cerevisiae*	4,982	151,766	60.93	0.38	2.09	4	0.01,223

Analysis of experimental evidence for PPIs indicated that over half of the interactions were identified by HT technologies (Fig. S1), implying that these technical advances have significantly advanced the construction and analysis of protein interactomes. Furthermore, the interaction network of all species, except for yeast, showed an approximately scale-free topological structure, with degree exponents ranging from 1.340 to 3.281 (Fig. S2). The comparative analysis of global network topological properties revealed that well-studied species (humans and yeast) exhibited higher average degrees, shorter average path lengths, and smaller diameters (Table 1).

### Estimation of human interactome size using six reference species

Observed variations in interactome sizes primarily reflect research bias among species. Here, we estimated the corresponding size of the human interactome using six reference species to enable the comparative analysis of network structural changes across species (see Methods for more details; Fig. 1B). The final estimated human interactome size varied between 9,093 (frog) and 458,595 (yeast) PPIs, encompassing proteins ranging from 6,116.460 ± 39.829 (frog) to 17,450.899 ± 17.375 (yeast) (Fig. 1C). These findings underscore significant heterogeneity in scientific investigation efforts among species.

### Variation in the local network structure offers a new framework for cross-species comparative disease research

**Fig 2:**
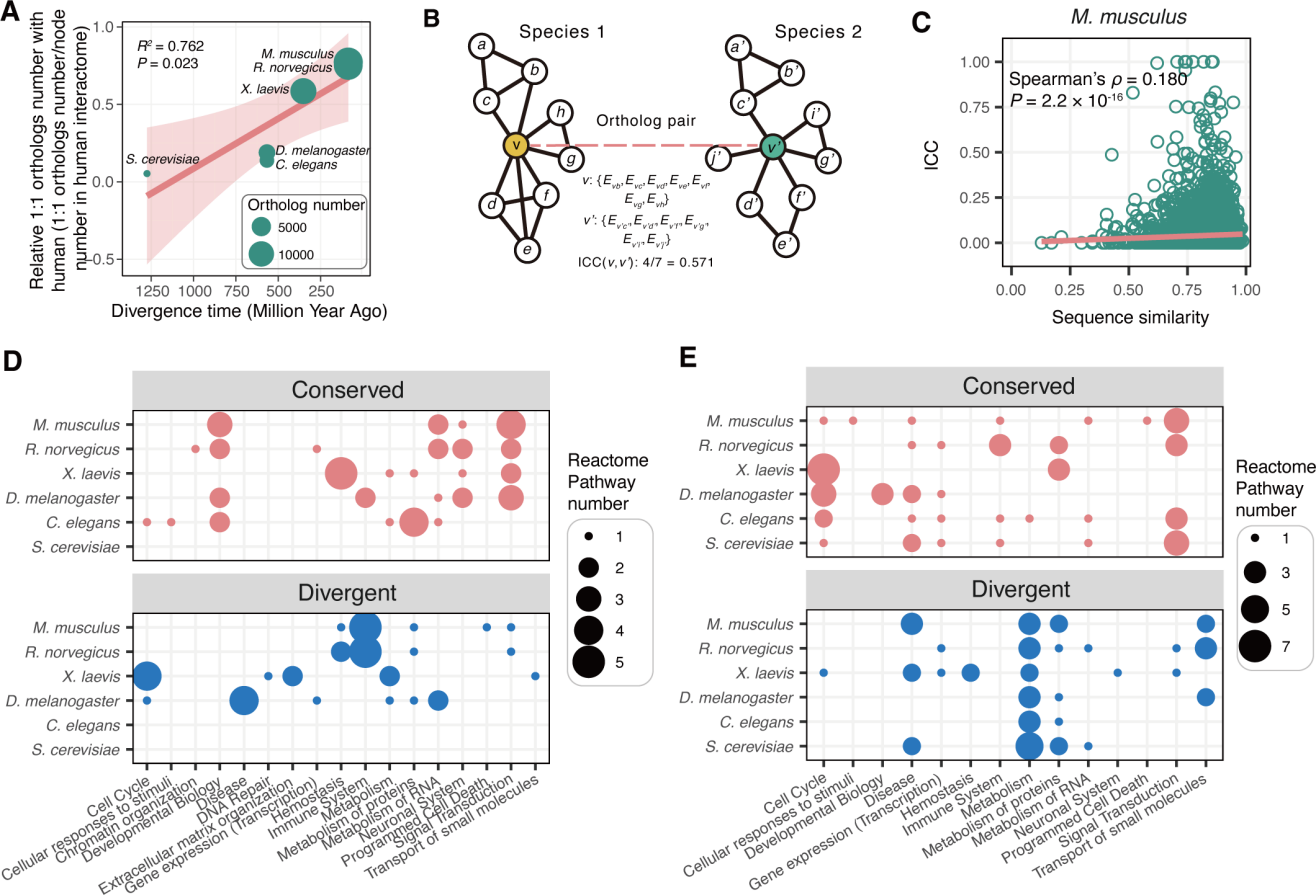
Two metrics for assessing the evolutionary conservation of proteins. (**A**) The relative number of 1:1 orthologous proteins between humans and other species. The point size represents the absolute number of 1:1 orthologs. (**B**) ICC measures the proportion of conserved PPIs between protein *v* in humans and its orthologous protein *v*'. (**C**) The correlation between ICC and sequence similarity in orthologous protein pairs between humans and mice. Statistical results of gene set enrichment analysis based on sequence similarity scores (**D**) and ICC values (**E**). Dot size represents the number of pathways within the enriched pathway categories. Red and blue colors indicate conserved and divergent pathways, respectively. ICC, interaction conservation coefficient.

The statistical analysis of protein-coding gene families revealed that genes within gene families predominantly exist in single-copy form, with proportions ranging from 78.12% (*C. elegans*) to 98.82% (human) (Fig. S3 B). The number of 1:1 orthologs across species was found to be inversely correlated with the divergence time from humans (*R*^2^ = 0.762, *P* = 0.023;, Fig. 2A), while only 605 of the 1:1 orthologs were shared among all seven species (Fig. S3 C and D). Only orthologous proteins included in the interactome were considered in this study.

We computed the interaction conservation coefficients (ICCs) for 1:1 orthologs between the human reference species and six other species to investigate conservation of the local network architecture (see Materials and Methods for details; Fig. 2B). We first analyzed the correlation between ICCs and sequence similarity in orthologous protein pairs and found no significant association between them (Spearman’s ≤ 1.6 <0.200; Fig. 2C; Fig. S4). Subsequently, we systematically ranked distinct orthologous protein sets using the six reference species based on ICC values and sequence similarity metrics to facilitate gene set enrichment analysis (GSEA). Our analyses revealed distinct evolutionary patterns at different biological scales. At the genetic level, orthologous proteins demonstrated significant conservation in pathways, including developmental biology and the neuronal system (Fig. 2D; Fig. S5). Conversely, at the local network level, these genes exhibited marked divergence in pathways such as the transport of small molecules and metabolism (Fig. 2E; Fig. S6). Notably, disease-related pathways displayed a dual pattern, showing both divergent and conserved evolutionary trends among orthologs (Fig. 2E; Fig. S6).

### The identification of viral modules is influenced by the size of the host interactome

**Fig 3:**
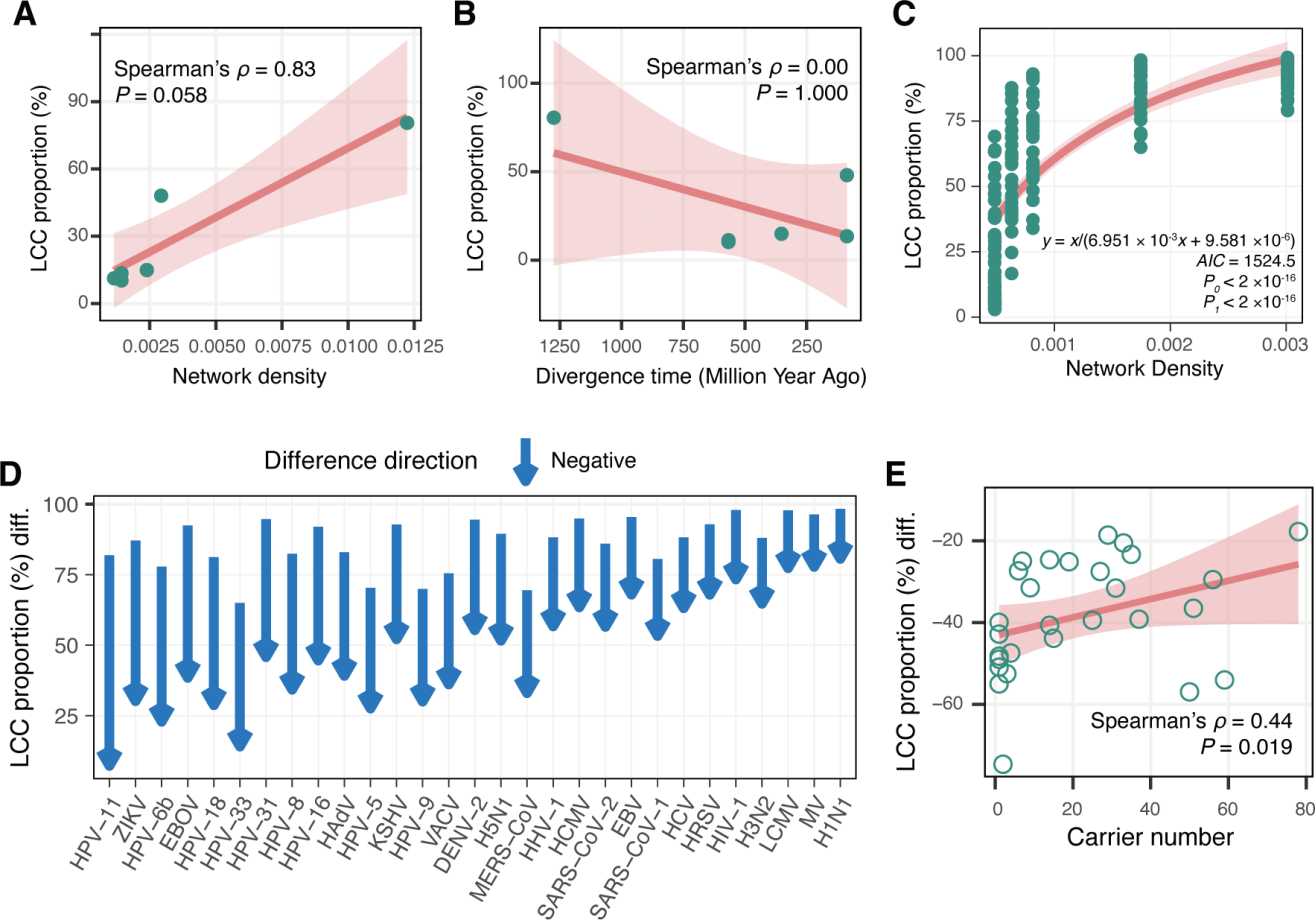
Heterogeneity in the LCC proportion across species. Correlation between LCC proportion and network density (**A**) and divergence time between six species and humans (**B**). (**C**) The linear relationship between the mean LCC proportion and network density for 6,000 randomized human interactomes. Lines show the generalized linear model of LCC proportion values, and the colored bands indicate a 95% confidence band for the linear fit. (**D**) Comparison of LCC proportions in the mouse interactome versus size-matched human interactomes. The blue vectors signify reduced connectivity conservation. (**E**) Correlation between host carrier number and LCC proportion differences for viruses. LCC, largest connected component; AIC, Akaike information criterion.

The cross-species analysis of network clustering patterns for viral VTPs (human interactome) and their orthologs (species-specific interactomes) enables the detection of evolutionarily conserved viral modules and mechanistic divergence in pathogenic strategies. Therefore, we characterized the size of the largest connected component (LCC) formed by VTPs (or their orthologous proteins) within their respective interactomes, as well as its proportion relative to all VTPs (Fig. S7). The LCC proportion was correlated with the network density of the host interactome (Spearman’s ≥ 0.05 = .830, *P* = 0.058; Fig. 3A); however, no association with species divergence time was found (Spearman’s ρ = 0.000, *P* = 1.000; Fig. 3B).

Furthermore, we systematically investigated the potential size-dependency of viral module identification within the human interactomes. We implemented a generalized linear model (GLM) with network density (randomly sampled network above) as the independent variable and the LCC proportion as the dependent variable. The fitted model demonstrated a significant monotonically increasing relationship between network density and LCC proportion (*y* = *x*/(6.951 × 10^−3^*x* + 9.581 × 10^−6^), AIC = 1,524.5, *P*_0_ < 2.0 × 10^−16^, *P*_1_ < 2.0 × 10^−16^; Fig. 3C), indicating enhanced viral module detectability in denser networks. Nevertheless, this trend exhibited asymptotic deceleration with increasing network density. This phenomenon was also consistently observed at the individual virus level (Fig. S8).

Laboratory mice have been the preeminent model organism in biomedical research for decades, owing to their genetic and physiological similarities to humans (14). Here, we compared the LCC proportion for 29 viruses between mice and 1,000 size-matched human interactomes. Our analysis revealed a consistent and significant reduction in viral LCC proportions within the mouse interactome relative to their human counterparts (permutation test, *Z* score < −1.6; Fig. 3D), with differences varying from −17.714% (H1N1) to −74.660% (HPV-11). The observed LCC proportion difference was positively correlated with the number of viral hosts (Spearman’s ρ = 0.440, *P* = 0.019; Fig. 3E).

Cross-species comparative analysis further revealed heterogeneity in LCC proportion disparities. For example, in comparisons involving *C. elegans* and flies versus humans, viruses such as HIV-1 and H1N1 displayed substantial variations in LCC proportions, whereas MERS-CoV and HPV-33 showed relatively minor differences (Fig. S9). Quantitative assessment demonstrated a systematic increase in LCC proportions for HPV strains and coronaviruses within rat and frog interactomes relative to human networks (LCC proportion difference > 0; Fig. S9), potentially reflecting size-dependent artifacts from reduced interactome sizes. Notably, our analysis revealed a positive correlation between LCC proportion differences (comparing the interactomes of mouse and yeast with that of humans) and the viral host loads (Spearman’s ρ > 0, *P* < 0.05; Fig. S10).

### The perturbation exerted by viruses on the local network architecture of the hosts exhibits a high degree of complexity

**Fig 4:**
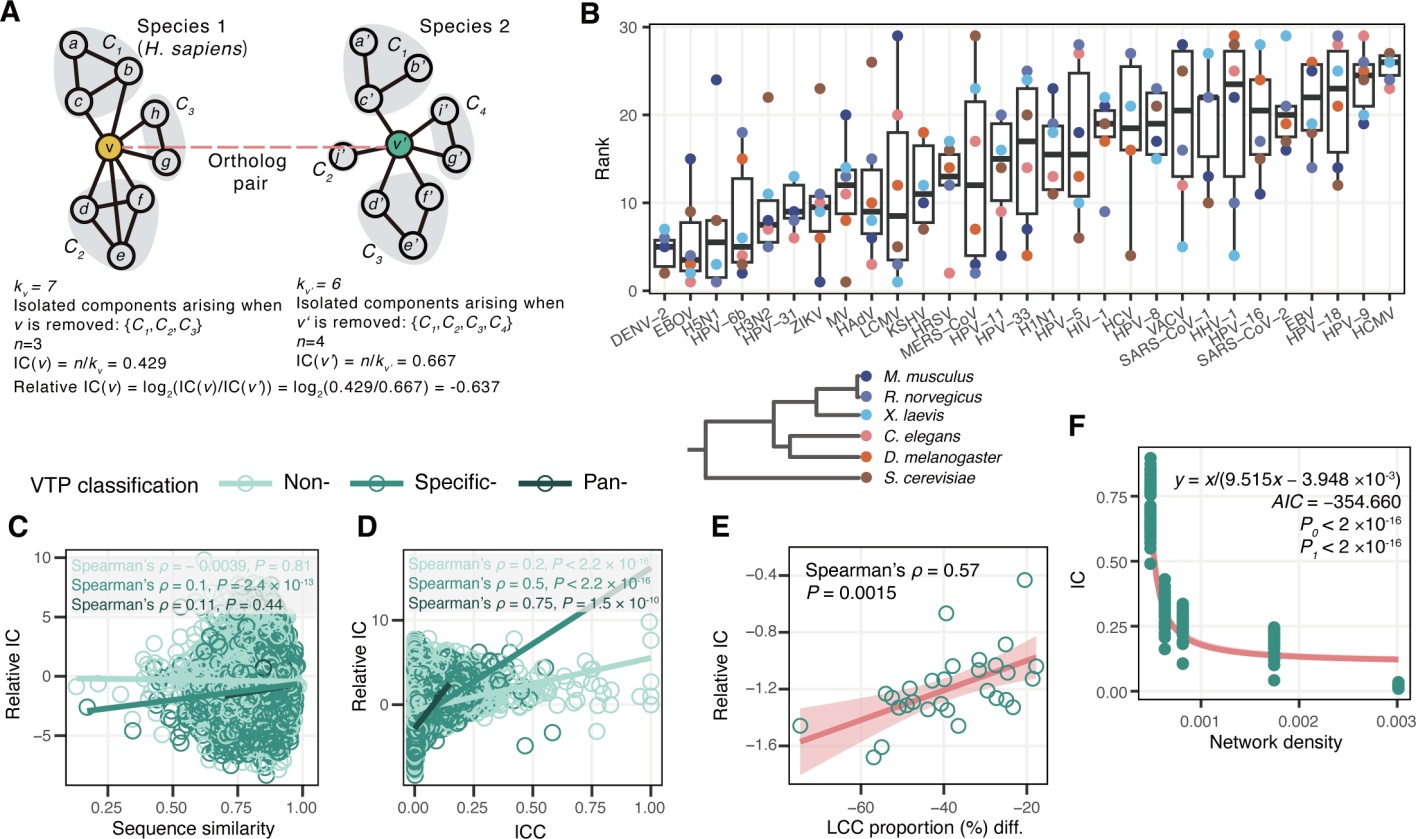
Perturbations exerted by viruses on host interactomes. (**A**) The relative IC measures the change in topological fragmentation between protein *v* in humans and its orthologous protein *v*'. (**B**) The ranking of viruses by mean relative IC values in each comparator species (in descending order), followed by aggregation of their average ranks across all six species. The correlation between relative IC and sequence similarity (**C**) and ICC (**D**) in orthologous gene pairs of three classifications (non-, specific-, and pan-VTP) between humans and mice. (**E**) The correlation between mean relative IC and LCC proportion differences for 29 viruses between humans and mice. (**F**) The linear relationship between the mean IC and network density for 6,000 randomized human interactomes. Lines show the generalized linear model of mean IC values, and the colored bands indicate a 95% confidence band for the linear fit. IC, isolated components; LCC, largest connected component; ICC, interaction conservation coefficient; AIC, Akaike information criterion.

The evolutionary acquisition of host interactome resilience likely arises through persistent host-pathogen interactions, with the manifestation of such adaptive robustness being observable through changes in the network structure of individual proteins. We adopted the relative fold change of Zitnik et al.’s isolated component (IC) metric (10) in orthologous VTPs across species interactomes to decipher how viral perturbations shape network resilience (see Materials and Methods for details; Fig. 4A). The comparative analysis of 29 viruses across six species (vs. size-matched human interactomes) showed that human VTPs had lower IC values than non-rat species orthologs (relative IC < 0; Fig. S11), indicating enhanced antiviral resilience in human interactomes compared to other species.

The ranking of viruses by mean relative IC values exhibited non-trivial cross-species complexity in their ordinal distribution (Fig. S11). We ranked viruses based on their mean relative IC values in each comparator species to investigate the patterns of viral perturbation across different host species (in descending order), followed by the aggregation of their average ranks across all six species. This analysis revealed DENV-2 as the top-ranked pathogen, indicating that it consistently exhibited the lowest mean relative IC values among all studied viruses (Fig. 4B). This pattern indicates that DENV-2 exerts stronger disruptive effects on host interactomes than other viruses, reflecting its broad-spectrum network perturbation capability across multiple species. In contrast, HCMV was ranked lowest, suggesting that its network perturbations in these six species may be comparable to or even weaker than its effects on the human interactome (Fig. 4B).

Next, we classified proteins into three categories, non-, specific-, and pan-VTP according to previous studies (15), and investigated potential factors influencing their relative IC values. We found no correlation between the relative IC values of the three protein categories and the sequence similarity of their protein-coding genes when comparing humans and other species (Spearman’s ρ ≤ 0.250; Fig. 4C; Fig. S12). Pan-VTPs showed a stronger correlation with ICC values than specific-VTPs, whereas no correlation relationship was observed for non-VTPs (Fig. 4D; Fig. S13). These findings suggest that it is the local network structure that is subject to selection during virus–host interactions, thereby contributing to the maintenance of network resilience. Moreover, the relative IC values were correlated with LCC proportion differences (Fig. 4E; Fig. S14), indicating that VTPs that are more likely to form viral modules tend to exhibit higher network resilience.

Then, we ranked the orthologous VTPs of 29 viruses between human and mouse by relative IC values and performed GSEA to identify differential Reactome pathways. The results showed that the differential pathways were primarily enriched in categories such as the immune system, metabolism, and signal transduction (Fig. S15). For HIV-1, the significantly enriched pathways included Interleukin-12 family signaling, Activation of the mRNA upon binding of the cap-binding complex and eIFs and subsequent binding to the 43S complex, and Response of EIF2AK4 (GCN2) to amino acid deficiency (Fig. S15). For H1N1, the significantly enriched pathways included the Innate Immune System, MHC class II antigen presentation, and Clathrin-mediated endocytosis, among others (Fig. S15).

Finally, the potential size-dependency of ICs within the human interactomes was systematically investigated. A GLM with network density (randomly sampled network above) was constructed as the independent variable and IC as the dependent variable. The fitted model revealed a significant monotonically decreasing relationship between network density and IC (*y* = *x*/(9.515*x* -3.948 × 10^−3^), *AIC* = -354.660, *P*_0_ < 2.0 × 10^−16^, *P*_1_ < 2.0 × 10^−16^; Fig. 4F), indicating reduced network fragmentation in denser networks. Nevertheless, this trend exhibited asymptotic deceleration with increasing network density. This phenomenon was also consistently observed at the individual virus level (Fig. S16).

### Network metrics are correlated with the approval rates of non-vaccine therapeutics

In disease research, the choice of animal model is a critical determinant of whether clinical therapeutics ultimately receive approval. Since most studies use mice as experimental models, we separately examined the correlations between differences in network and genetic metrics between humans and mice and the probabilities of progressing from phase 1 trials to approval (PoS_1A_) in vaccine and non-vaccine anti-infective drug development programs. We categorized 28 viruses into 11 categories according to the disease classification of Lo et al. (16), and extracted the PoS_1A_ for each category (Table S2).

**Fig 5:**
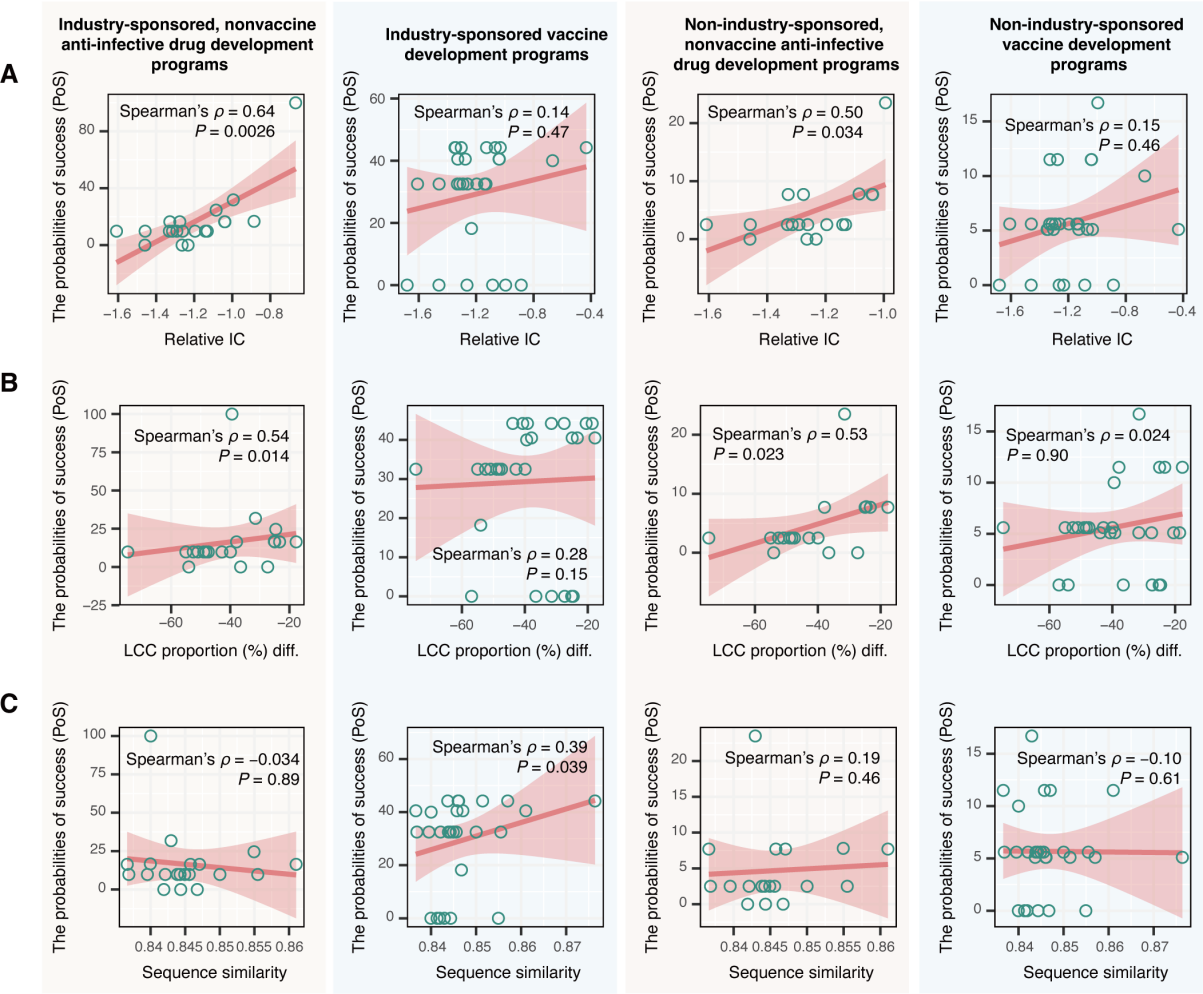
The correlation between network metrics and the approval rates of antiviral therapeutics. The correlation between mean relative IC (**A**), LCC proportion differences (**B**), and sequence similarity (**C**) and the probabilities of success from phase 1 trials to approval (PoS_1A_) of vaccine and non-vaccine anti-infective drug development programs. IC, isolated components; LCC, largest connected component.

The correlations analysis revealed that network metrics (including LCC proportion differences and relative IC) were generally more strongly associated with the PoS_1A_ of non-vaccine drug programs (Fig. 5A and B), with Spearman’s *ρ* ranging from 0.50 (*P* = 0.034) to 0.64 (*P* = 0.0026). Conversely, Spearman’s *ρ* between network metrics and the PoS_1A_ of vaccine programs were all below 0.300 (*P* > 0.100; Fig. 5A and B). Moreover, the PoS_1A_ of industry-sponsored programs showed stronger correlations with network metrics (Fig. 5A and B). No significant correlations were observed between genetic metrics (namely sequence similarity) and PoS_1A_ (Spearman’s ρ < 0.400, *P* > 0.040; Fig. 5C).

## DISCUSSION

In this study, we applied a network biology framework to explore cross-species virus-host interaction patterns. We found that the viral module size and the extent of viral perturbations to local network structures were correlated with the interactome size (i.e., network density). Although host-specific perturbations were more complex than those observed in humans, viral targets generally exhibited negative relative IC values. Moreover, relative IC values were correlated with ICCs and were positively associated with the non-vaccine therapeutic success rates for infectious viral diseases. This analysis offers a systematic approach to evaluating the applicability of animal models in infectious viral disease research.

### Assessment of network size and its impact on virus-host interaction research

Advances in high-throughput interactomics have enabled the systematic exploration of complex disease mechanisms. The primary goal of this study is to explore, from an evolutionary perspective, the differences in network structures that respond to the same external perturbation across species, rather than focusing mainly on host immune responses or drug reactions. Therefore, we selected species based on two main criteria: (i) high-quality, genome-wide interactomes (including at least 1,000 PPIs) available for model species, and (ii) coverage of a broad evolutionary range (from yeast to human) to distinguish conserved network behaviors from species-specific adaptations. A key question remains: how large must an interactome be to sufficiently elucidate complex disease phenotypes? Menche et al. demonstrated that disease modules can be reliably detected in incomplete human interactomes when ≥25 disease-associated genes are involved (17). However, they did not assess how interactome size affects the identification of the same disease module, limiting assessment of network completeness. Prior research estimated the human interactome to range between 150,000 and 370,000 PPIs (10, 18–20). By integrating available data, we obtained networks ranging from 1,528 PPIs in frogs to 698,482 PPIs in humans. Analyses showed a linear relationship between network density and both LCC proportion and IC values: as density increased, viral modules expanded, while individual node contributions to stability decreased, with this effect plateauing at high densities. The GLM results also revealed that local network disruption upon viral target removal decreases with increasing density, with an elbow point at the human network sampled from *D. melanogaster*. Yeast, a well-studied model with high network density, showed smaller differences from human LCC and IC values. Experimental methods may detect PPIs absent under natural conditions, and simple data integration may include context-specific “snapshot” interactions, both affecting network-based analyses. In rat-human comparisons, some viruses showed positive LCC proportion differences and relative IC values, likely due to small-network discrepancies. Expanding beyond traditional models toward biomedically relevant species would strategically advance interactomics and disease research.

### Viral perturbation and resilience of the host network

Resilience, defined as a system’s ability to recover from perturbations, is a fundamental property of biological systems (21). Biological systems comprise functional units ranging from macromolecules to species that interact through networks and display structural features such as complexity, robustness, and dynamic behavior (22). Network science offers quantitative frameworks to assess robustness, particularly through analyses of node failure. Two main scenarios describe network failures (23): random failures, in which real-world networks are generally robust due to heterogeneous connectivity, and targeted attacks, where removing critical nodes leads to collapse. Zitnik et al. showed that interactomes become more resilient during evolution, indicating increasing robustness to network failures over time (10). Klein et al. extended this by adding proteins using different strategies, finding that gene expression-based preferential attachment preserved or enhanced interactome resilience across species (24). Constrained by small genomes, viruses rely on host processes to complete their life cycle and act as external perturbations that can shape host resilience. Our previous work showed that viruses preferentially target human proteins with high degree and centrality rather than interacting randomly (15). By comparing viral perturbations from an evolutionary perspective, this study revealed that viruses exerted a smaller impact on the human interactome. This suggests that the human network has evolved enhanced resilience through long-term virus-host “arms races,” even when viruses target central network nodes.

Following infection, viruses can persist for months to over a year, as seen with HPV (25) and SARS-CoV-2 (26). We assessed network resilience via node-removal strategy; however, viral proteins integrate into host interactomes and the effects of such node additions remain understudied. Ackerman et al. constructed virus-host integrated networks for influenza A and SARS-CoV-2, identifying proteins that modulate cellular behavior and represent potential therapeutic targets (27, 28). Comparative cross-species virus-integrated network frameworks may uncover differences in system stability and host cellular responses to viral perturbations.

### The strengths and limitations of network metrics

Complex biological phenotypes often involve multiple gene pathways, making pathway-level divergence or conservation signals more prevalent than gene-level signals (29). For example, mapping mutated genes onto biomolecular networks to identify shared pathways is an effective strategy for interpreting rare mutations in tumors (30, 31). Here, we used IC values to compare viral perturbations across species and found that the relative IC values of VTPs, especially pan-VTPs, correlated with ICCs. These findings suggest that these proteins preserve their structural context and are less prone to network failure upon viral infection than specific- and non-VTPs. Additionally, relative IC values and LCC proportion differences were positively related to the approval rates of non-vaccine therapeutics, indicating that interspecies network differences may predict translational success. Using the relative IC values between human and mouse for GSEA, we found that the pathways were mainly enriched in the immune system, metabolism, and signal transduction. For example, the enrichment of pathways related to IL-12 family signaling, cap-dependent translation initiation, and GCN2-mediated amino acid deficiency response in HIV-1-infected hosts reveals critical species-specific differences between humans and preclinical animal models (e.g., mice). These pathways govern key aspects of antiviral immunity and viral protein synthesis. However, their activation thresholds, signal dynamics, and viral evasion mechanisms may differ substantially between species. Consequently, interventions that appear effective in mouse models may fail in clinical trials, or vice versa, leading to potential misjudgments of preclinical efficacy. No correlation was observed between network metrics and vaccine approval rates, likely because vaccine efficacy depends on immune responses, including immune gene expression and antibody repertoire dynamics, which are not captured by the VTP network structure alone.

In summary, we established a cross-species network comparison framework to investigate virus-host interactions and provide theoretical guidance for selecting appropriate animal models in infectious disease research. However, certain limitations must be addressed in future work. On one hand, our findings were derived from an evolutionary systems biological view based on seven selected species. Therefore, the conclusions may not be directly generalizable to other widely used preclinical models, particularly non-human primates or zoonotic species. Direct extrapolation to other models would require dedicated interactome mapping and perturbation experiments using cells or tissues from those species. On the other hand, the limited number of data sets (only 29 viruses) and the exclusive use of network features are insufficient for developing a predictive framework for estimating translational therapeutic success. Further integration of AI-driven multimodal data sets beyond interactomes (32) will be necessary to better dissect and predict the outcomes of viral infection.

## MATERIALS AND METHODS

### Data collection

#### Protein interactome data

The PPIs of different species were extracted from five primary source databases: BioGRID (33), DIP (34), IntAct (35), MINT (36), and MatrixDB (37). Additionally, a data set investigating the interactomes across mouse tissues (38) was also included. Seven species with the highest whole-genome coverage, namely human, mouse, rat, African clawed frog, fruit fly, *C. elegans*, and yeast, were ultimately selected as the study subjects.

For each reported PPI, the interacting proteins were converted to gene symbol pairs using an ID mapping table downloaded from Ensembl (http://asia.ensembl.org) (39) or Xenbase (https://www.xenbase.org). Interactions lacking valid PubMed IDs (PMIDs) or PSI-MI methods (40, 41), genetic or protein-DNA interactions, and PPIs annotated with “invalid” evidence terms (42, 43) were excluded. As in our previous study (15), PPIs were classified into high-throughput (HT) and low-throughput (LT) subsets based on the size of the data set in the publication.

#### Orthologs and species tree

Homologous gene family data were retrieved from the HomoloGene database on the NCBI (https://ftp.ncbi.nih.gov/pub/HomoloGene/build68). Among these data, genes derived from a common ancestral gene and those retaining similar functions across different species were referred to as orthologs (44). If these orthologs existed as only one single copy in each species, they were classified as 1:1 orthologs.

The species tree and its corresponding divergence times were obtained from TimeTree 5 (http://timetree.org) (45). Species silhouette images were downloaded from PHYLOPIC (https://www.phylopic.org). The tree visualization was implemented using the R package ggtree (46).

#### Virus-human PPIs

Protein interactions between the 29 most reported viruses and human cells were obtained from our previous study (15). The VTPs on the human interactome were retained for further analysis. Furthermore, the viral targets were ranked in descending order based on the number of viruses by which they were targeted. The top 1% of these genes—each targeted by at least 14 viruses—were defined as pan-VTPs, while the remainder were classified as specific-VTPs. Our previous computational framework (47) was employed to analyze cross-species VTP network distributions, focusing on LCCs in species-specific interactomes as indicators of viral modules and evolutionary divergence.

#### Viral host ranges

The host carrier information for 29 viruses was extracted from the ENHanCEd Infectious Diseases (EID2) database (https://eid2.liverpool.ac.uk/).

#### The probabilities of success from phase 1 trials to the approval (PoS_1A_) of vaccines and other anti-infective therapeutics

The PoS_1A_ of clinical trials for vaccines and other anti-infective therapeutics from January 2000 to December 2019, covering 2,544 vaccine and 6,829 non-vaccine programs, was obtained from Lo et al. (16). As the COVID-19 pandemic began in early 2020, the PoS_1A_ for SARS-CoV-2 was not evaluated. Programs were classified as industry- or non-industry-sponsored based on commercial involvement. A total of 1,838 industry-sponsored vaccines, 706 non-industry vaccines, 3,851 industry non-vaccine drugs, and 2,978 non-industry non-vaccine drugs were found.

#### Human reactome pathway data

In total, 1,615 human reactome pathways and their associated genes were retrieved from MSigDB (48). These pathways were categorized into 29 classes based on information from https://reactome.org, with pathway categories containing more than 2,000 genes selected for further analysis.

### Sequence similarity analysis

Coding sequences (CDSs) of genes from seven species were retrieved from Ensembl and Xenbase databases (https://download.xenbase.org), and the longest transcript CDS per gene was selected. 1:1 orthologous gene pairs were identified between each species and humans, and their sequences were aligned pairwise using MAFFT v7.520 (49, 50). Alignments were trimmed using trimAl v1.5.rev0 (51). Pairwise genetic distances were estimated using the TN93 model (52) and the dist.dna() function in the R package ape v5.8-1 (53). Sequence similarity was calculated as 1 − genetic distance.

### Network topological properties analysis

The global network topological properties—including the mean degree, average clustering coefficient, mean shortest path length, and network diameter—were calculated using the NetworkX module (54) in Python. Network density, an important metric that describes the degree of connectivity in a network and reflects the proportion of actual connections to the maximum possible number of connections, was calculated as $density=\;\frac{2M}{N\left(N-1\right)}$, where *N* and *M* are the number of nodes and edges.

### Estimation of the human interactome size

The estimation of human interactome size comprised four steps. First, *M_ref_* and *N_ref_* were taken, and the probability *p_ref_* of each possible edge/PPI in the reference species was computed as *p*_ref_ = *M*_ref_/$\left(\begin{array}{c} N_{ref}\\ 2 \end{array}\right)$. Second, the expected mean degree in the reference species interactome was calculated as *A*_ref_ = *N*_*ref*_ *p*_ref_. Third, the expected mean degree and the average interaction rewiring rate of an individual edge quantified by Zitnik et al. (10) were used to calculate the expected mean degree *A_h_* in the human interactome as: *A_h_* = 2^IRR^*A*_ref_. The interaction rewiring rate (IRR) was calculated using the following equation:


$$IRR\left(i;\;v,\;v^{\prime }\right)\;=\;\log_{2}\frac{i\left(v\right)}{i\left(v^{\prime }\right)}$$

where *v* and *v*′ are the proteins whose neighborhoods were compared. Here, given two proteins from different species, *v* was selected as the protein from the organism with more nucleotide substitutions per site. When “frog” was used as the reference species, an IRR of 0.215 was applied to estimate the human interactome size, as frogs exhibit a higher average evolutionary rate than humans (*t*_frog_ = 4.012, *t*_human_ = 3.997). In contrast, for the other five reference species, an IRR of −0.215 was used. The resulting *A_h_* was then used to compute the probability *p_h_* of each possible edge/PPI in humans as follows: *p_h_* = *A_h_*/*N_h_*. Finally, *M_h_* was obtained as *M_h_* = (Nh2)*p_h_*.

Six species were used as references to estimate the edge number (*M_s_*) in the human interactome corresponding to *N_s_* = 18,101 nodes and ensure cross-species network comparability. Starting from *M_s_*, a degree-weighted random sampling of edges was conducted from the original human interactome, with a step size of −100, i.e., extracting *M_s_* – 100*n* edges at each iteration, where *n =* 0, 1, 2, … denotes the number of iterations. To estimate the corresponding theoretical edge count *M_e_*, the node number (*N_e_*) within the largest connected component was recorded after disconnected nodes were removed. This iterative process continued until *M_e_ > M_s_ –* 100*n*. At this point, the current edge count *M_e_* was designated as the final edge number in the human interactome, denoted *M_h_*. Afterward, *M_h_* edges were randomly sampled from the original human interactome, and the number of nodes (*N_h_*) in the largest connected component was recorded after excluding disconnected nodes. This randomization procedure was repeated 1,000 times for each reference species, yielding 6,000 randomized human interactomes.

### Network comparison between humans and other species

#### Interaction conservation coefficient (ICC)

The IRR can only quantitatively assess the interaction rewiring; it does not reflect the degree of conservation within interactions. Therefore, an ICC was used to assess the rewiring conservation:


$$ICC\left(i;\;v,\;v^{\prime }\right)\;=\;\frac{n}{i\left(v\right)}$$

$$n=i\left(v\right)\cap i\left(v^{\prime }\right)$$

where *n* denotes the number of orthologous protein pairs present among the neighbors of proteins *v* and *v*′ in their respective interactomes. ICC values range from 0 to 1, with values near one indicating higher conservation of the local network environment around the node *v*, whereas values approaching 0 reflect lower conservation.

#### Isolated components (ICs)

ICs are defined as the number of connected components that arise when the central node *v* is removed from the neighborhood and it is normalized by *v*’s degree:


$$IC\left(v\right)=\frac{n}{k_{v}}$$

where $n=\left|\left\{C_{1},C_{2},\ldots ,C_{n}\right\}\right|$ represents the number of isolated network components after *v* is removed, and *k_v_* represents node *v*’s degree in the original network. The metric takes values in [1/max*_v_ k_v_*, 1] and a higher value indicates a greater fragmentation of the network neighborhood.

A relative fold-change metric was employed to facilitate the comparative analysis of IC values between human protein *v* and its orthologous counterparts *v*' across species, defined as follows:


$$relative\;IC(v)\;={log}_{2}\frac{\frac{1}{n}\sum_{i=1}^{n}\;{IC}_{i(v)}}{IC(v^{\prime })}$$

where *n* denotes the number of randomized human interactomes (see above). A relative IC value greater than zero reflects increased topological fragmentation in protein *v*’s network neighborhood compared to its ortholog *v*′. Conversely, a negative relative IC value signifies reduced network fragmentation relative to the orthologous counterpart. Values approximating zero indicate stronger evolutionary conservation of interconnectivity patterns within the local network neighborhoods of the proteins.

### Gene set enrichment analysis

Orthologs between each species and humans ranked by ICC and sequence similarity values were used to identify conserved and divergent Reactome pathways. The enrichment analysis was performed using the GSEA() function in the clusterProfiler package with default parameters, except that nPermSimple = 100,000 was used to handle ties in the preranked statistics (55). Pathways with Benjamini–Hochberg-adjusted *P*-value <0.01 (56) were considered significantly enriched.

### Statistical analysis

Spearman’s correlation coefficient was performed using the stat_cor() function in the ggpubr package. Generalized linear models were fitted with the stat_poly_eq() function from the ggpmisc package. Parameter estimation was conducted using the glm() function with *y* ~ *I*(1/*x*) and family = gaussian(link = "inverse").

## Data Availability

The source code and related data are available on GitHub (https://github.com/TangLab-GDMU/VHI_divergence.git).

## SUPPLEMENTAL MATERIAL

Supplemental material mbio.00195-26-s0001.docx Supplemental figures and tables.

ASM does not own the copyrights to Supplemental Material that may be linked to, or accessed through, an article. The authors have granted ASM a non-exclusive, world-wide license to publish the Supplemental Material files. Please contact the corresponding author directly for reuse.
