# Supplementary material for: Divergence in virus-host protein interactomes across species explains disparities in translational therapeutic success
Source: mBio. 2026 Jun 22;17(8):e00195-26. doi: 10.1128/mbio.00195-26 (PMC13463867; doi:10.1128/mbio.00195-26)
Supplement: Supplementary file 1 [file mbio.00195-26-s0001.docx]

**Supplementary materials**

**Supplementary Table 1.** Collection and integration of PPI datasets for seven species

**Supplementary Table 2.** The probabilities of success from phase 1 to approval (PoS_1A_) of vaccine and nonvaccine anti-infective drug development programs.

**Supplementary Figure 1.** The supporting evidences for each PPI in integrated PPIN for *H. sapiens*, *M. musculus*, *R. norvegicus*, *X. laevis*, *D. melanogaster*, *C. elegans*, and *S. cerevisiae*.

**Supplementary Figure 2.** Power-law degree distribution of protein interactomes for *H. sapiens*, *M. musculus*, *R. norvegicus*, *X. laevis*, *D. melanogaster*, *C. elegans*, and *S. cerevisiae*.

**Supplementary Figure 3.** Orthologs among seven species.

**Supplementary Figure 4.** The correlation between ICC and sequence similarity in orthologous gene pairs between *H. sapiens* and other six species.

**Supplementary Figure 5.** Gene set enrichment analysis of orthologs based on sequence similarity scores.

**Supplementary Figure 6.** Gene set enrichment analysis of orthologs based on ICC values.

**Supplementary Figure 7.** LCC size and its proportion to all viral targets on interactomes of seven species.

**Supplementary Figure 8.** The linear relationship between the LCC proportation and network density.

**Supplementary Figure 9.** Comparison of LCC proportion in six species interactomes versus 1000 scale-matched human interactomes.

**Supplementary Figure 10.** The correlation between the host carrier number and LCC proportion differences for viruses.

**Supplementary Figure 11.** Comparison of IC values of VTGs in the 1000 scale-matched human interactomes against their orthologs’ IC values in interactomes of six species.

**Supplementary Figure 12.** The correlation between relative IC and sequence similarity in orthologous gene pairs of three classifications (non-, specific- and pan-VTP) between *H. sapiens* and other six species.

**Supplementary Figure 13.** The correlation between relative IC and ICC in orthologous gene pairs of three classifications (non-, specific- and pan-VTP) between *H. sapiens* and other six species.

**Supplementary Figure 14.** The correlation between mean relative IC and LCC proportion differences for 29 viruses between *H. sapiens* and other six species.

**Supplementary Figure 15.** Gene set enrichment analysis of orthologous VTPs of 29 viruses between human and mouse based on relative IC values.

**Supplementary Figure 16.** The linear relationship between the mean IC and network density.

**Supplementary Table 1. Collection and integration of PPI datasets for seven species**

| Species | Dataset source | | | | | | Network size | |
| --- | --- | --- | --- | --- | --- | --- | --- | --- |
|  | BioGRID | DIP | IntAct | MINT | MatrixDB | Skinnider et al. 2021 | Number of nodes *N* | Number of edges *M* |
| *H. sapiens* | √ | √ | √ | √ | √ |  | 18,101 | 698,482 |
| *M. musculus* | √ | √ | √ | √ |  | √ | 11,362 | 187,121 |
| *R. norvegicus* | √ | √ | √ |  |  |  | 2,937 | 5,828 |
| *X. laevis* | √ |  | √ |  |  |  | 1,234 | 1,594 |
| *D. melanogaster* | √ | √ | √ | √ |  |  | 8,933 | 56,303 |
| *C. elegans* | √ | √ | √ |  |  |  | 7,776 | 32,567 |
| *S. cerevisiae* | √ | √ | √ | √ |  |  | 4,982 | 151,766 |

**Supplementary Table 2. The probabilities of success (%) from phase 1 to approval (PoS_1A_) of vaccine and nonvaccine anti-infective drug development programs.**

| Virus | Disease | Vaccine development programs | | Nonvaccine anti-infective drug development programs | | |
| --- | --- | --- | --- | --- | --- | --- |
|  |  | Industry-sponsored | Non-industry-sponsored | | Industry-sponsored | Non-industry-sponsored |
| DENV-2 | Others | 44.2 | 5.1 | | / | / |
| EBOV | Ebola | 18.2 | 0 | | 0 | 0 |
| EBV | Others | 44.2 | 5.1 | | / | / |
| HCV | Hepatitis C Virus (HCV) | 0 | 0 | | 16.7 | / |
| HAdV | Others | 44.2 | 5.1 | | / | / |
| HCMV | Cytomegalovirus (CMV) Infection | 0 | 16.7 | | 31.8 | 23.5 |
| HHV-1 | Others | 44.2 | 5.1 | | / | / |
| HIV-1 | Human Immunodeficiency Virus (HIV) | 0 | 0 | | 24.6 | 7.8 |
| HPV-5 | Human Papillomavirus (HPV) | 32.5 | 5.6 | | 9.8 | 2.5 |
| HPV-6b | Human Papillomavirus (HPV) | 32.5 | 5.6 | | 9.8 | 2.5 |
| HPV-8 | Human Papillomavirus (HPV) | 32.5 | 5.6 | | 9.8 | 2.5 |
| HPV-9 | Human Papillomavirus (HPV) | 32.5 | 5.6 | | 9.8 | 2.5 |
| HPV-11 | Human Papillomavirus (HPV) | 32.5 | 5.6 | | 9.8 | 2.5 |
| HPV-16 | Human Papillomavirus (HPV) | 32.5 | 5.6 | | 9.8 | 2.5 |
| HPV-18 | Human Papillomavirus (HPV) | 32.5 | 5.6 | | 9.8 | 2.5 |
| HPV-31 | Human Papillomavirus (HPV) | 32.5 | 5.6 | | 9.8 | 2.5 |
| HPV-33 | Human Papillomavirus (HPV) | 32.5 | 5.6 | | 9.8 | 2.5 |
| HRSV | Respiratory Infections | 40.5 | 11.5 | | 16.4 | 7.7 |
| H1N1 | Respiratory Infections | 40.5 | 11.5 | | 16.4 | 7.7 |
| H3N2 | Respiratory Infections | 40.5 | 11.5 | | 16.4 | 7.7 |
| H5N1 | Respiratory Infections | 40.5 | 11.5 | | 16.4 | 7.7 |
| KSHV | Others | 44.2 | 5.1 | | / | / |
| LCMV | Others | 44.2 | 5.1 | | / | / |
| MV | Others | 44.2 | 5.1 | | / | / |
| MERS-CoV | Middle East Respiratory Syndrome (MERS) | 0 | 0 | | 0 | 0 |
| SARS-CoV-1 | Severe Acute Respiratory Syndrome (SARS) | 0 | 0 | | 0 | 0 |
| SARS-CoV-2 | / | / | / | | / | / |
| VACV | Smallpox | 40 | 10 | | 100 | / |
| ZIKV | Zika | 0 | 0 | | / | / |

**Supplementary Figure 1.** The supporting evidences for each PPI in integrated PPIN for *H. sapiens*, *M. musculus*, *R. norvegicus*, *X. laevis*, *D. melanogaster*, *C. elegans*, and *S. cerevisiae* (A-G). Green, blue and orange sectors represent HT, HT;LT and LT, respectively. HT, High-Throughput; LT, Low-Throughput.

**Supplementary Figure 2**. Power-law degree distribution of protein interactomes for *H. sapiens*, *M. musculus*, *R. norvegicus*, *X. laevis*, *D. melanogaster*, *C. elegans*, and *S. cerevisiae* (A-G).

**Supplementary Figure 3**. Orthologs among seven species. (A) Species tree. (B) Homologous gene family sizes. (C) Number of 1:1 orthologs across species. (D) Number of 1:1 orthologs between H. sapiens and other species.

**Supplementary Figure 4**. The correlation between ICC and sequence similarity in orthologous gene pairs between H. sapiens and other six species. ICC, interaction conservation coefficient.

**Supplementary Figure 5**. Gene set enrichment analysis of orthologs based on sequence similarity scores. For each species, only the top 10 most conserved (denoted in red, NES > 0) and most divergent (denoted in blue, NES < 0) enriched pathways were visualized here. NES, normalized enrichment score.

**Supplementary Figure 6**. Gene set enrichment analysis of orthologs based on ICC values. For each species, only the top 10 most conserved (denoted in red, NES > 0) and most divergent (denoted in blue, NES < 0) enriched pathways were visualized here. NES, normalized enrichment score; ICC, interaction conservation coefficient.

**Supplementary Figure 7**. LCC size and its proportion to all viral targets on interactomes of seven species. LCC, largest connected component; DENV-2, Dengue virus 2; EBOV, Ebola virus; EBV, Epstein-Barr virus; HAdV, Human adenovirus C; HCMV, Human cytomegalovirus; HCV, Hepatitis C virus; HHV-1, Human herpesvirus 1; HIV-1, Human immunodeficiency virus 1; HPV-5, HPV-6b, HPV-8, HPV-9, HPV-11, HPV16, HPV-18, HPV-31 and HPV-33, Human papillomavirus 5, 6b, 8, 9, 11, 16, 18, 31 and 33; HRSV, Human respiratory syncytial virus; H1N1, H3N2 and H5N1, Influenza A H1N1, H3N2 and H5N1 virus; KSHV, Kaposi’s sarcoma-associated herpesvirus; LCMV, Lymphocytic choriomeningitis virus; MV, Measles virus; MERS-CoV, Middle East respiratory syndrome coronavirus; SARS-CoV-1, severe acute respiratory syndrome coronavirus 1; SARS-CoV-2, severe acute respiratory syndrome coronavirus 2; VACV, Vaccinia virus; ZIKV, Zika virus.

**Supplementary Figure 8**. The linear relationship between the LCC proportation and network density. Lines show the generalized linear model of LCC proportation values; color bands indicate 95% confidence band for the linear fit. LCC, largest connected component; AIC, Akaike information criterion.

**Supplementary Figure 9**. Comparison of LCC proportion in six species interactomes versus 1000 size-matched human interactomes. Directional indicators represent interspecies LCC proportion differences. Blue vectors signify reduced connectivity conservation, whereas red vectors indicate enhanced module retention. Z score $\leq\left| 1.6 \right|$ (P value $\geq0.05$) was considered to indicate statistically non-significant differences. LCC, largest connected component.

**Supplementary Figure 10**. The correlation between the host carrier number and LCC proportion differences for viruses. LCC, largest connected component.

**Supplementary Figure 11**. Comparison of IC values of VTGs in the 1000 scale-matched human interactomes against their orthologs’ IC values in interactomes of six species. Blue dots indicate that the fragmentation level of the human interactome after removing VTGs is lower than that of the comparator species' interactomes under the same perturbation, while red dots represent the opposite trend. *** P-value < 0.001, ** P-value < 0.01, * P-value < 0.05, ns, not significantly different. IC, isolated components.

**Supplementary Figure 12**. The correlation between relative IC and sequence similarity in orthologous gene pairs of three classifications (non-, specific- and pan-VTP) between H. sapiens and other six species. IC, isolated components.

**Supplementary Figure 13**. The correlation between relative IC and ICC in orthologous gene pairs of three classifications (non-, specific- and pan-VTP) between H. sapiens and other six species. IC, isolated components; ICC, interaction conservation coefficient.

**Supplementary Figure 14**. The correlation between mean relative IC and LCC proportion differences for 29 viruses between H. sapiens and other six species. IC, isolated components; LCC, largest connected component.

**Supplementary Figure 15**. Gene set enrichment analysis of orthologous VTPs of 29 viruses between human and mouse based on relative IC values.

**Supplementary Figure 16**. The linear relationship between the mean IC and network density. Lines show the generalized linear model of mean IC values; color bands indicate 95% confidence band for the linear fit. IC, isolated components; AIC, Akaike information criterion.
